# Supplementary material for: No increased risk of cancer associated with metal-on-metal or ceramic-on-ceramic procedures compared to other bearing surfaces in patients with total hip arthroplasty: A nationwide linked registry cohort analysis of 167,837 patients
Source: PLoS One. 2022 Nov 30;17(11):e0278241. doi: 10.1371/journal.pone.0278241 (PMC9710777; doi:10.1371/journal.pone.0278241)
Supplement: S1 Table — Abbreviation: ICD-10 = International Classification of Diseases 10th Revision. (DOCX) [file pone.0278241.s001.docx]

**Supporting information**

**S1 Table: ICD codes included in analysis.**

| **Cancer site** | **ICD-10 Codes and Description** |
| --- | --- |
| **Prostate** | **C61 Malignant neoplasm of prostate** |
| **Melanoma** | **C43 Malignant melanoma of skin** |
| **Non-Hodgkins** | **C82 Follicular lymphoma** **C83 Mature T/NK-cell lymphomas** **C84 Non-follicular lymphoma**  **C85 Other and unspecified types of non-Hodgkin lymphoma** **C86 Other specified types of T/NK-cell lymphoma** |
| **Myeloma** | **C90 Multiple myeloma and malignant plasma cell neoplasms** |
| **Leukaemia** | **C91 Lymphoid leukaemia****C92 Myeloid leukaemia****C93 Myeloid leukaemia****C94 Other leukaemias of specified cell type****C95 Leukaemia of unspecified cell type** |
| **Lung** | **C33 Malignant neoplasm of trachea** **C34 Malignant neoplasm of bronchus and lung** |
| **Colon** | **C18 Malignant neoplasm of colon** |
| **Bladder** | **C67 Malignant neoplasm of bladder** |
| **Kidney** | **C64 Malignant neoplasm of kidney, except renal pelvis** |
| **Bone** | **C40 Malignant neoplasm of bone and articular cartilage of limbs****C41 Malignant neoplasm of bone and articular cartilage of other and unspecified sites** |

Abbreviation: ICD-10 = International Classification of Diseases 10th Revision.
