# Supplementary material for: No increased risk of cancer associated with metal-on-metal or ceramic-on-ceramic procedures compared to other bearing surfaces in patients with total hip arthroplasty: A nationwide linked registry cohort analysis of 167,837 patients
Source: PLoS One. 2022 Nov 30;17(11):e0278241. doi: 10.1371/journal.pone.0278241 (PMC9710777; doi:10.1371/journal.pone.0278241)
Supplement: Attachment — Submitted filename: REVISIONE ARTICOLO Protesi danca.docx [file pone.0278241.s002.docx]

**Paper Title**:

*No increased risk of cancer after total hip arthroplasty associated with metal-on-metal hip arthroplasty: a nationwide linked registry cohort analysis of 167,837 patients*

**Reviewer comments**

This observational retrospective study aims to evaluate the risk of all-cause and site-specific cancer in patients undergoing total hip arthroplasty and, in particular, when using Metal-on-Metal bearing surfaces. To this end data from the Australian Orthopaedic Association National Joint Replacement Registry (AOANJRR) have been linked to data collected by the Australasian Association of Cancer Registries.

This is in an interesting study and the findings presented may add knowledge on the topic. Anyway, some major and minor revisions are required to make it eligible for publication.

**Major revisions:**

Title:

- As the findings presented in the manuscript highlight no increased risk of cancer associated to MoM procedures as compared to other prosthesis (MoP, CoP), and not to the general population, should the Authors consider rephrasing the title?

Methods section:

- Page 5, Line 90: Authors state that “the AOANJRR has recorded all hip and knee joint replacement procedures from 1 September 1999, becoming completely national by mid-2002.” Did Authors consider not to include in the analyses the joint replacement procedures in this time window to prevent any potential selection bias?

- When selecting patients for the study, it would recommend to better explain how Authors were able to assess that the procedures recorded in the AOANJRR registry were related only to patients with osteoarthritis and not with other pathologies that may require hip replacement.

**Minor revisions:**

Methods section:

- Page 6, line 109: the acronyms “AACR” and “AIHW” are for?

- Page 6, line 116: the acronym “ACD” is for?

- I would recommend deleting the subsection title for “Australian orthopaedic association national joint replacement registry” and “Australian cancer database”. Consider to remove “Data linkage” as well, while I would suggest to change “Participants” into “Study population”.

- Page 7, Statistical analysis: What about mentioning the descriptive statistics presented in Table 1?

- Page 7, Line 135: Please refer to the calculation of 95% Confidence Intervals also.

Results section:

- Page 9, Line 161: Check the number of csTHA procedures (156.516 instead of 165.516?)

- S1 Table: Insert the meaning of the acronyms.

- Page 9, Line 169: a reference symbol to the note at the bottom is not present within the table

- Table 3: “SIR” and “CI” is for?

-Table 4: “HR” and “SIR” stand for?
